# Climbing Escher’s stairs: A way to approximate stability landscapes in multidimensional systems

**DOI:** 10.1371/journal.pcbi.1007788

**Published:** 2020-04-10

**Authors:** Pablo Rodríguez-Sánchez, Egbert H. van Nes, Marten Scheffer

**Affiliations:** Department of Aquatic Ecology and Water Quality Management, Wageningen University, Wageningen, The Netherlands; University of Illinois at Urbana-Champaign, UNITED STATES

## Abstract

Stability landscapes are useful for understanding the properties of dynamical systems. These landscapes can be calculated from the system’s dynamical equations using the physical concept of scalar potential. Unfortunately, it is well known that for most systems with two or more state variables such potentials do not exist. Here we use an analogy with art to provide an accessible explanation of why this happens and briefly review some of the possible alternatives. Additionally, we introduce a novel and simple computational tool that implements one of those solutions: the decomposition of the differential equations into a gradient term, that has an associated potential, and a non-gradient term, that lacks it. In regions of the state space where the magnitude of the non-gradient term is small compared to the gradient part, we use the gradient term to approximate the potential as quasi-potential. The non-gradient to gradient ratio can be used to estimate the local error introduced by our approximation. Both the algorithm and a ready-to-use implementation in the form of an R package are provided.

## Author summary

The physical concept of potential, also referred to as stability landscape, has proved to be a useful visualization tool for explaining and communicating advanced concepts about dynamical systems to professional communities as diverse as environmental scientists, ecologists, economists, mathematicians and policy makers. It is a particularly popular tool in cell biology, where it is often referred to as Waddington’s epigenetic landscape. Unfortunately, for most biological models with more than one interacting element, such a stability landscape doesn’t exist unless the system under study satisfies very restrictive conditions. Here we use an analogy with art to provide an accessible explanation of the subtle reasons a potential may fail to exist for a given biological system. Additionally, we introduce a remarkably simple and efficient algorithm to compute potentials that takes into account these limitations, providing the best quasi-potential candidate plus an error map. This error map tells us under which conditions can we trust the potential picture.

This is a *PLOS Computational Biology* Methods paper.

## Introduction

With knowledge becoming progressively more interdisciplinary, the relevance of science communication is rapidly increasing. Mathematical concepts are among the hardest topics to communicate to non-expert audiences, policy makers, and also to scientists with little mathematical background. Visual methods are known to be successful ways of explaining mathematical concepts and results to non-specialists.

**Fig 1:**
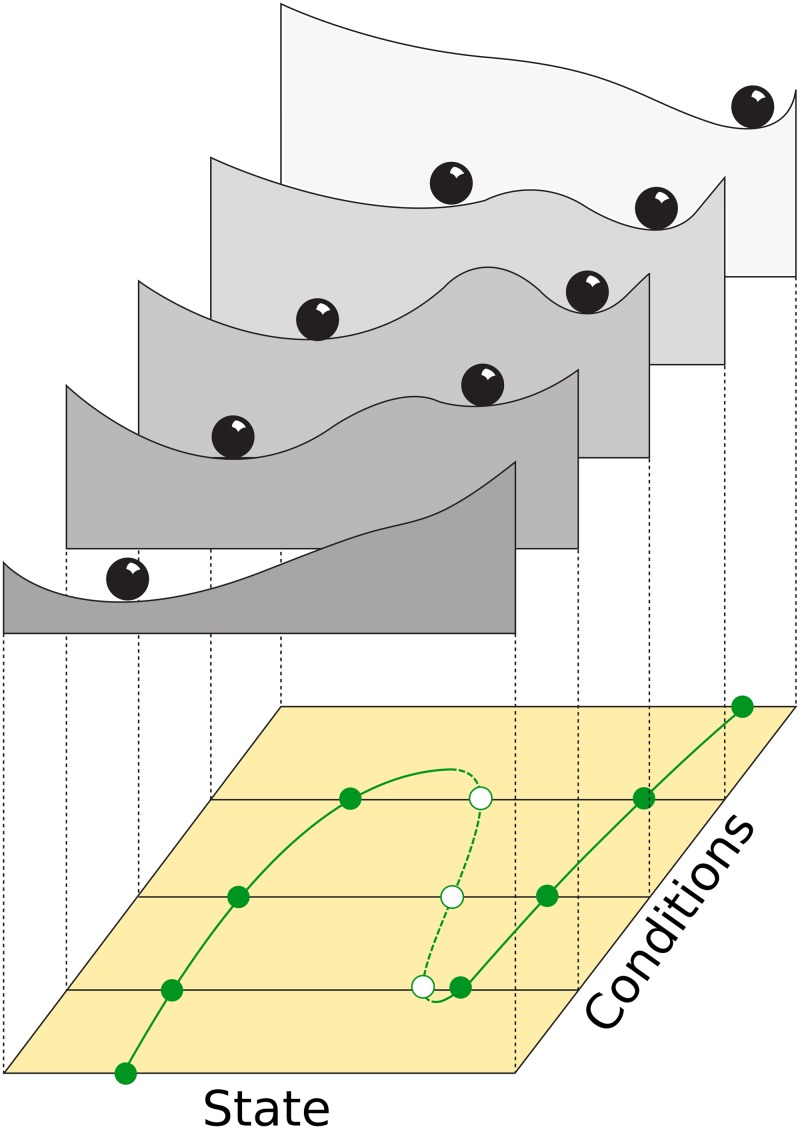
Example of a set of 5 stability landscapes used to illustrate bistability in ecosystems (e.g: Forest/Desert, eutrophicated lake/clear water, etc., see [7]). The upper side of the figure shows the stability landscape of a one-dimensional system for 5 different values of a control parameter. The lower side shows the bifurcation diagram, where the filled points represent stable equilibria and the empty points unstable ones. This diagram proved to be a successful tool for explaining advanced concepts in dynamical systems theory such as bistability and fold bifurcations to scientific communities as diverse as ecologists, mathematicians and environmental scientists.

One particularly successful visualization method is that of the stability landscape, also known as scalar potential, Waddington’s epigenetic landscape, rolling marble diagram or ball-in-a-cup diagram [1–5]. In the rest of this work we define the stability landscape as a classical scalar potential, and thus we will use both terms as equivalents (for a precise mathematical definition, see subsection “Mathematical background” below). In stability landscapes (e.g.: Fig 1) the horizontal position of the marble represents the state of the system at a given time. With this picture in mind, the shape of the surface represents the underlying dynamical rules, where the slope is proportional to the speed of the movement. The peaks on the undulated surface represent unstable equilibrium states and the wells represent stable equilibria. Different basins of attraction are thus separated by peaks in the surface. Stability landscapes, whose origin can be traced back to the introduction of the scalar potential in physics by Lagrange in the 18th century [6], have proven to be a successful tool to explain advanced concepts about dynamical systems theory in an intuitive way. Some examples of those advanced concepts are multistability, basin of attraction, bifurcation points and hysteresis (see [7], [3] and Fig 1).

The main reason for the success of this picture arises from the fact that stability landscapes are built as an analogy with our most familiar dynamical system: movement. Particularly, the movement of a marble along a curved landscape under the influence of its own weight. The stability landscape corresponds then with the physical concept of potential energy [2]. This explains why our intuition, based in what we know about movement in our everyday life, works so well reading these type of diagrams. It is important to stress the fact that under this picture there’s not such a thing as inertia [4]. The accurate analogy is that of a marble rolling in a surface while submerged inside a very viscous fluid [2].

**Fig 2:**
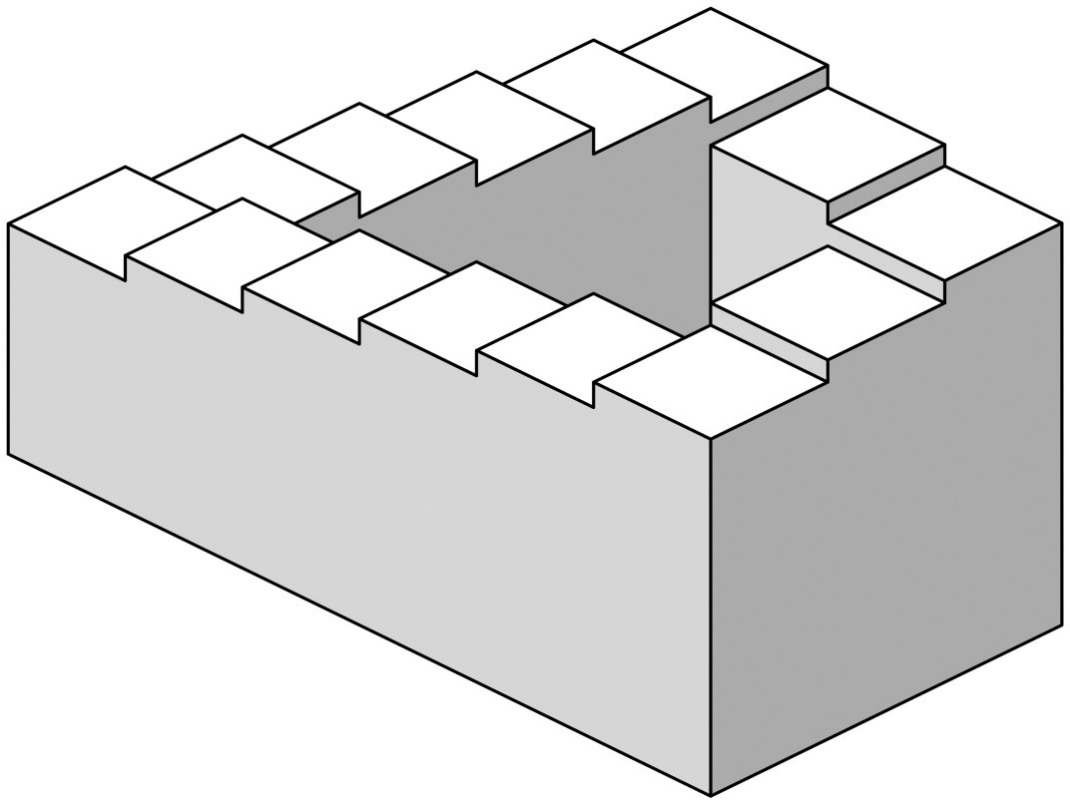
The Penrose stair [8] is a classical example of an impossible object. In such a surface, it is possible to walk in a closed loop while permanently going downhill. The scalar potential of a system with a cyclic attractor, if existed, should have the same impossible geometry. This object was popularized by the Dutch artist M.C. Escher (for two beautiful examples, see [9] and [10]).

Like with any other analogy, it is important to be aware of its limitations. The one we address here is the fact that, for models with more than one state variable, such a potential doesn’t exist in general. To get an intuitive feeling of why this is true, picture a model with a stable cyclic attractor. As the slope of the potential should reflect the speed of change, we would need a potential landscape where our marble can roll in a closed loop while always going downhill. Such a surface is a classical example of an impossible object (see Fig 2 and [8] for details).

As this is a centuries-old problem, it is perhaps not surprising that several methods have been proposed to approximate stability landscapes for general, high-dimensional systems. Helmholtz, in his pioneering work on fluid dynamics in the 19th century, decomposes the dynamical equations into a gradient and a curl term [11, 12]. Helmholtz uses the gradient term to compute a well defined scalar potential. The curl term cannot be associated to a scalar potential, and requires computing a more complicated mathematical object, namely a vector potential. Vector potentials, although useful in fluid dynamics and electromagnetism (see for instance chapters 5 and 16 of [13]), do not correspond with the idea of a stability landscape. A similar approach is followed in the normal decomposition [12], where the dynamical equations are decomposed into two perpendicular directions, being one of them the gradient of a potential and the other interpreted as a perpendicular force. Another possible decomposition was introduced by Ao [14, 15], consisting in applying the sum of a symmetric and an antisymmetric linear transformation to the dynamical equations. Some interesting alternatives are presented in [4], like potentials for second-order systems or the use of Lyapunov functions as stability landscapes.

Alternative approaches based on probabilistic considerations have achieved great success. The underlying idea is that those states who are more stable have a higher chance to be found in the state space. The corresponding quasi-potential is then a function of the probability density function associated with the stochastic model. This is the approach followed by the Wang’s potential landscape [16] and by the Freidlin-Wentzell potential [17] (a good review comparing both can be found in [12]). Particularly, the Freidlin-Wentzell potential has become the standard for the derivation of quasi-potentials of stochastic systems. Its direct links with transition rates makes it particularly useful to understand and visualize relative stabilities [18, 19]. Other quasi-potential approaches, and even the exact potential corresponding to a stochastic differential equation with a purely gradient deterministic part, are just limit cases of it [12]. It is important to note that these probability-based quasi-potentials don’t admit, in general, the straightforward interpretation of a “rolling marble”. Additionaly, they require heavy mathematical weaponry, such as partial and stochastic differential equations. For a complete review about this topic, please refer to [12].

In the present work we introduce a simple method to deal with the fundamental problem of approximating stability landscapes for high dimensional deterministic systems. Specificially, we introduce an algorithm to easily perform an approximation of the above-mentioned Helmholtz decomposition, i.e., to decompose differential equations as the sum of a gradient and a non-gradient, divergence-free part. Each part can be used, respectively, to compute an associated scalar potential and to measure the local error introduced by our picture. In order to reach those interested readers with little background in mathematics, we limited our mathematical weaponry. Knowledge of basic linear algebra and calculus will suffice to understand the paper to its last detail. Additionally, we provide a ready to use, tested and documented R package that implements the algorithm this paper describes [20].

### Mathematical background

#### Conditions of the potential to exist

Consider a coupled differential equation with two state variables *x* and *y* (Eq (1)).

$$\left\{ \begin{array}{c} \frac{dx}{dt}=f\left(x,y\right)\\ \frac{dy}{dt}=g\left(x,y\right) \end{array}\right.$$ (1)

If, in addition, we are able to find a two-dimensional function *V*(*x*, *y*) whose slope is proportional to the change in time of both states, then *V* represents the stability landscape of the system (see Eq (2), and compare it with (1)).

$$\left\{ \begin{array}{c} \frac{dx}{dt}=f\left(x,y\right)=-\frac{\partial V(x,y)}{\partial x}\\ \frac{dy}{dt}=g\left(x,y\right)=-\frac{\partial V(x,y)}{\partial y} \end{array}\right.$$ (2)

It can be shown that such a function *V*(*x*, *y*) only exists if the crossed derivatives of functions *f*(*x*, *y*) and *g*(*x*, *y*) are equal for all *x* and *y* (Eq (3)). Systems satisfying Eq (3) are known as conservative, irrotational or gradient fields (cf. section 8.3 of [21]). Function *V* is known as scalar potential in the physical and mathematical literature.

$$\begin{array}{c} \frac{\partial f}{\partial y}=\frac{\partial g}{\partial x} \end{array}$$ (3)

If condition (3) holds we can use a line integral ([21], section 7.2) to invert (2) and calculate *V*(*x*, *y*) using the functions *f*(*x*, *y*) and *g*(*x*, *y*) as an input. An example of this inversion is Eq (4), where we have chosen an integration path composed of a horizontal and a vertical line.

$$\begin{array}{c} V\left(x,y\right)=V\left(x_{0},y_{0}\right)-\int_{x_{0}}^{x}f\left(\xi ,y_{0}\right)d\xi -\int_{y_{0}}^{y}g\left(x,\eta \right)d\eta \end{array}$$ (4)

The attentive reader may have raised her or his eyebrow after reading the word *chosen* applied to an algorithm. In fact, we can introduce this arbitrary choice without affecting the final result. The condition for potentials to exist (3) implies that any line integral between two points in this vector field should be independent of the path (cf. section 7.2 of [21]). Going back to our rolling marble analogy, we can gain some intuition about why this is true: in a landscape the difference in potential energy between two points is proportional to the difference in height, and thus stay the same for any path. If the condition (3) is not fulfilled, the potential calculated with (4) will depend on the chosen integration path. As this is an arbitrary choice, the computed potential will be an artifact with no natural meaning.

A generalization to more dimensions of these ideas is presented in the supplementary material S1 Text, available online.

#### Linearization of dynamical systems

It is known that under very general circumstances (particularly, local differentiability), a dynamical system can be approximated in the vicinity of a point (*x*_0_, *y*_0_) by using a first order Taylor expansion (Eq (5)). This process is known as linearization (cf. section 6.3 of [2]).

$$\left\{ \begin{array}{c} f\left(x,y\right)\approx f\left(x_{0},y_{0}\right)+\frac{\partial f(x_{0},y_{0})}{\partial x}\left(x-x_{0}\right)+\frac{\partial f(x_{0},y_{0})}{\partial y}\left(y-y_{0}\right)\\ g\left(x,y\right)\approx g\left(x_{0},y_{0}\right)+\frac{\partial g(x_{0},y_{0})}{\partial x}\left(x-x_{0}\right)+\frac{\partial g(x_{0},y_{0})}{\partial y}\left(y-y_{0}\right) \end{array}\right.$$ (5)

Eq (5) can be written more compactly in matrix form (see Eq (6)), where the square matrix contains the partial derivatives evaluated at the point (*x*_0_, *y*_0_). This matrix is known as the Jacobian *J*(*x*_0_, *y*_0_).

$$\begin{array}{c} \left[\begin{array}{c} f(x,y)\\ g(x,y) \end{array}]\approx [\begin{array}{c} f(x_{0},y_{0})\\ g(x_{0},y_{0}) \end{array}]+{\left[\begin{array}{cc} \frac{\partial f}{\partial x} & \frac{\partial f}{\partial y}\\ \frac{\partial g}{\partial x} & \frac{\partial g}{\partial y} \end{array}\right]}_{\left(x_{0},y_{0}\right)}\cdot [\begin{array}{c} x-x_{0}\\ y-y_{0} \end{array}\right] \end{array}$$ (6)

It is easy to see that condition (3) is equivalent to requiring the Jacobian matrix in Eq (6) to be symmetric at all linearization points (*x*_0_, *y*_0_).

#### A few concepts about square matrices

In the rest of this work we will use a few concepts from basic linear algebra. Here we briefly review them, for the convenience of the reader.

The transpose of a matrix is obtained by exchanging rows and columns or, equivalently, by “mirroring” it around its diagonal (see Eq (7) for an example).

$$\begin{array}{c} {\left[\begin{array}{cc} a & b\\ c & d \end{array}\right]}^{T}=[\begin{array}{cc} a & c\\ b & d \end{array}] \end{array}$$ (7)

A symmetric matrix is equal to its transpose (see matrix *S* in (8)). A skew-symmetric matrix is equal to minus its transpose (see matrix *K* in (8)). The diagonal elements of a skew-symmetric matrix are always zero.

$$\begin{array}{c} S=[\begin{array}{cc} a & b\\ b & d \end{array}]\;K=[\begin{array}{cc} 0 & b\\ -b & 0 \end{array}] \end{array}$$ (8)

A basic result from linear algebra states that any square matrix can be univocally expressed as the sum of a symmetric and a skew symmetric matrix. Particularly, the symmetric and skew parts are given by Eq (9).

$$\begin{array}{c} M=M_{symm}+M_{skew}\;\text{where:}\;\{\begin{array}{cc} M_{symm} & =\frac{1}{2}(M+M^{T})\\ M_{skew} & =\frac{1}{2}(M-M^{T}) \end{array} \end{array}$$ (9)

## Methods

For the sake of a compact and easy to generalize notation, in the rest of this paper we will arrange the equations of our system as a column vector (Eq (10) shows an example for a two-dimensional system).

$$\begin{array}{c} {}[\begin{array}{c} f(x,y)\\ g(x,y) \end{array}]=\vec{f}\left(\vec{x}\right) \end{array}$$ (10)

The method for deriving a potential we propose is based on Helmholtz’s idea of decomposing a vector field in a conservative or gradient part and a non-gradient part (see Eq (11)).

$$\begin{array}{c} \vec{f}\left(\vec{x}\right)={\vec{f}}_{g}\left(\vec{x}\right)+{\vec{f}}_{ng}\left(\vec{x}\right) \end{array}$$ (11)

The gradient term ${\vec{f}}_{g}(\vec{x})$ captures the part of the system that can be associated to a potential function, while the non-gradient term ${\vec{f}}_{ng}(\vec{x})$ represents the deviation from this ideal case. We’ll use ${\vec{f}}_{g}(\vec{x})$ to compute an approximate or quasi-potential. The absolute error of this approach is given as the euclidean size of the non-gradient term $|{\vec{f}}_{ng}(\vec{x})|$. In regions where the gradient term is stronger than the non-gradient term, the condition (3) will be approximately fulfilled, and thus the calculated quasi-potential will represent an acceptable approximation of the underlying dynamics. Otherwise, the non-gradient term is too dominant to approximate a potential landscape.

In order to achieve a decomposition like (11), we begin by linearizing our model equations. Any sufficiently smooth and continuous vector field $\vec{f}(\vec{x})$ can be approximated around a point $\vec{x_{0}}$ using Eq (12), where $J(\vec{x_{0}})$ is the Jacobian matrix evaluated at the point $\vec{x_{0}}$ and $\Delta \vec{x}$ is defined as the distance to this point, that is, $\Delta \vec{x}=\vec{x}-\vec{x_{0}}$, written as a column vector. Note that Eq (12) is just the generalized version of the two dimensional case shown in (6).

$$\begin{array}{c} \vec{f}\left(\vec{x}\right)\approx \vec{f}\left(\vec{x_{0}}\right)+J\left(\vec{x_{0}}\right)\Delta \vec{x} \end{array}$$ (12)

As usual in linearization, we have neglected the terms of order 2 and higher in Eq (12). This approximation is valid for $\vec{x}$ close to $\vec{x_{0}}$.

Using the skew-symmetric decomposition described in Eq (9), we can rewrite the Jacobian as in Eq (13):

$$\begin{array}{c} J=J_{symm}+J_{skew} \end{array}$$ (13)

When inserted in Eq (12) it becomes (14):

$$\begin{array}{c} \vec{f}\left(\vec{x}\right)\approx \vec{f}\left(\vec{x_{0}}\right)+J_{symm}\left(\vec{x_{0}}\right)\Delta \vec{x}+J_{skew}\left(\vec{x_{0}}\right)\Delta \vec{x} \end{array}$$ (14)

The first two terms in the left hand side of Eq (14) represent a gradient system, being the third and last term the only non-gradient term in the equation (see “Mathematical background”). Eq (14) represents thus a natural, well-defined and operational way of writing our vector field $\vec{f}(\vec{x})$ decomposed as in Eq (11) (see (15)).

$$\left\{ \begin{array}{cc} {\vec{f}}_{g}\left(\vec{x}\right) & \approx \vec{f}\left(\vec{x_{0}}\right)+J_{symm}\left(\vec{x_{0}}\right)\Delta \vec{x}\\ {\vec{f}}_{ng}\left(\vec{x}\right) & \approx J_{skew}\left(\vec{x_{0}}\right)\Delta \vec{x} \end{array}\right.$$ (15)

The non-gradient term ${\vec{f}}_{ng}(\vec{x})$ is a divergence-free field (see [21] and/or supplementary material S1 Text), so our proposed decomposition is an approximation of the Helmholtz decomposition [12] (in that sense, our decomposition is more akin to that of Wang [16] than to Ao’s [14], as may be wrongly suggested by the fact that Ao also uses the concepts of symmetry / anti-symmetry). The gradient term ${\vec{f}}_{g}(\vec{x})$ can thus be associated to a potential $V(\vec{x})$. This potential can be computed analytically for this linearized model using a line integral (see Eq (4) for the two dimensional case, or the supplementary material S1 Text for the general one). The result of this integration yields an analytical expression for the potential difference between the reference point $\vec{x_{0}}$ and another point $\vec{x_{1}}\equiv \vec{x_{0}}+\Delta \vec{x}$ separated by a distance $\Delta \vec{x}$ (see Eq (16)).

$$\begin{array}{c} \Delta V\left(\vec{x_{1}},\vec{x_{0}}\right)\equiv V\left(\vec{x_{1}}\right)-V\left(\vec{x_{0}}\right)\approx -\vec{f}\left({\vec{x}}_{0}\right)\cdot \Delta \vec{x}-\frac{1}{2}\Delta {\vec{x}}^{T}J_{symm}\left(\vec{x_{0}}\right)\Delta \vec{x} \end{array}$$ (16)

Provided we know the value of the potential at one point ${\vec{x}}_{previous}$, Eq (16) allows us to estimate the potential at a different point ${\vec{x}}_{next}$ (cf.: Eq (17)).

$$\begin{array}{c} V\left({\vec{x}}_{next}\right)\approx V\left({\vec{x}}_{previous}\right)+\Delta V\left({\vec{x}}_{next},{\vec{x}}_{previous}\right) \end{array}$$ (17)

**Fig 3:**
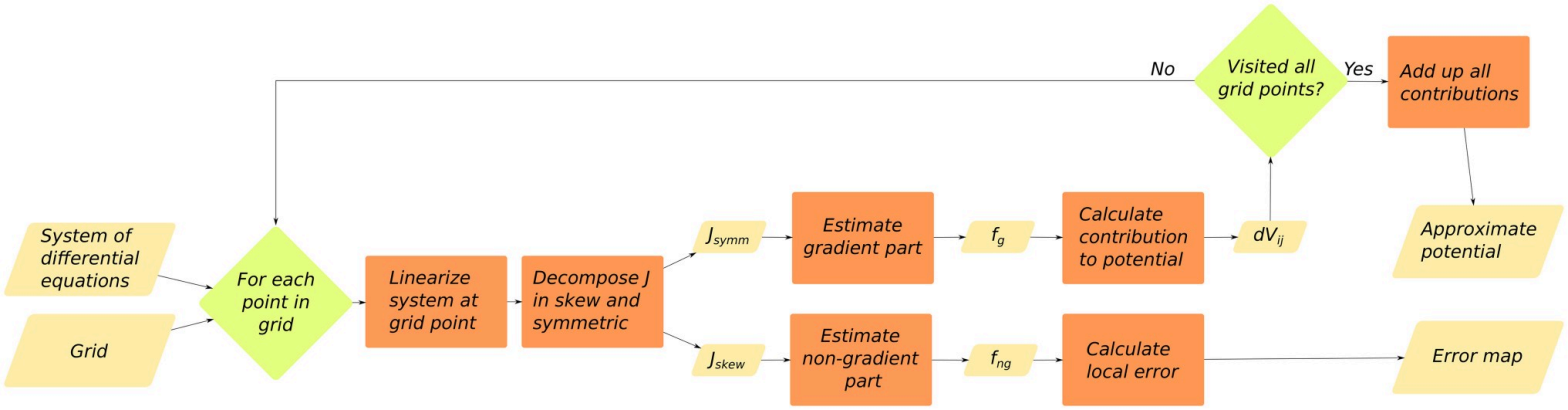
Flowchart showing the basic functioning of our implementation of the algorithm described in this paper.

Eq (17) can be applied sequentially over a grid of points to calculate the approximate potential on each of them. In two dimensions, the resulting potential is given by the closed formula (18). The cases with 3 and more dimensions can be generalized straightforwardly. For a step by step example, please refer to supplementary material S1 Text. For a flowchart overview of the algorithm, please refer to Fig 3.

$$\begin{array}{c} V\left(x_{i},y_{j}\right)=V\left(x_{0},y_{0}\right)+\sum_{k=1}^{i}\Delta V\left(x_{k},y_{0};x_{k-1},y_{0}\right)+\sum_{l=1}^{j}\Delta V\left(x_{i},y_{l};x_{i},y_{l-1}\right) \end{array}$$ (18)

As with any other approximation we need a way to estimate and control its error. The stability landscape described in (16) has two main sources of errors:

1. It has been derived from a set of linearized equations, sampled over a grid
2. It completely neglects the effects of the non-gradient part of the system

The error due to linearization in Eq (14) is roughly proportional to $|\Delta \vec{x}{|}^{2}$, where $|\Delta \vec{x}|$ is the euclidean distance to the reference point. By introducing a grid, we expect the linearization error to decrease with the grid’s step size.

The more fundamental error due to neglecting the non-gradient component of our system cannot be avoided by reducing the grid’s step choice. From Eq (11) it is apparent that the absolute error of our decomposition is given by $\vec{f}(\vec{x})-{\vec{f}}_{g}(\vec{x})={\vec{f}}_{ng}(\vec{x})$. That is, we can use the euclidean norm of ${\vec{f}}_{ng}(\vec{x})$ as an approximation of the local absolute error introduced by our algorithm. The relative error introduced by our approximation can be estimated using Eq (19), where we use Eq (15) to relate with the skew and symmetric parts of the Jacobian matrix.

$$\begin{array}{c} err\left(\vec{x}\right)\approx \frac{|{\vec{f}}_{ng}\left(\vec{x}\right)|}{|{\vec{f}}_{g}\left(\vec{x}\right)|+|{\vec{f}}_{ng}\left(\vec{x}\right)|}\approx \frac{|J_{skew}\left(\vec{x}\right)|}{|J_{symm}\left(\vec{x}\right)|+|J_{skew}\left(\vec{x}\right)|} \end{array}$$ (19)

The norms of the symmetric and skew Jacobians can be understood as the “weights” of each of those matrices. The relative error described in (19) quantifies how dominant the non-gradient term is in each region of the phase space, being 0 in those regions where the system is fully gradient (i.e.: ∣*J*_*skew*_∣ = 0), and 1 where it is fully non-gradient (i.e.: ∣*J*_*symm*_∣ = 0). The figures in the Results section show that the error maps calculated with Eq (19) are a good estimator of the local quality of our quasi-potential approximation.

### Implementation

As an application of the above-mentioned ideas, and following the spirit of reproducible research, we developed a transparent *R* package we called *rolldown* [20]. Our algorithm accepts a set of dynamical equations and a grid of points defining our region of interest as an input. The output is the estimated potential and the estimated error, both of them calculated at each point of our grid (see Fig 3 for details).

## Results

### Synthetic examples

#### A four well potential

We first tested our algorithm with a synthetic model of two uncoupled state variables. Uncoupled systems are always gradient as all non-diagonal values of the Jacobian are zero everywhere. We added the interaction terms *p*_*x*_ and *p*_*y*_ to be able to make it gradually non-gradient (see Eq (20)).

$$\left\{ \begin{array}{c} \frac{dx}{dt}=f\left(x,y\right)=-x\left(x^{2}-1\right)+p_{x}\left(x,y\right)\\ \frac{dy}{dt}=g\left(x,y\right)=-y\left(y^{2}-1\right)+p_{y}\left(x,y\right) \end{array}\right.$$ (20)

**Fig 4:**
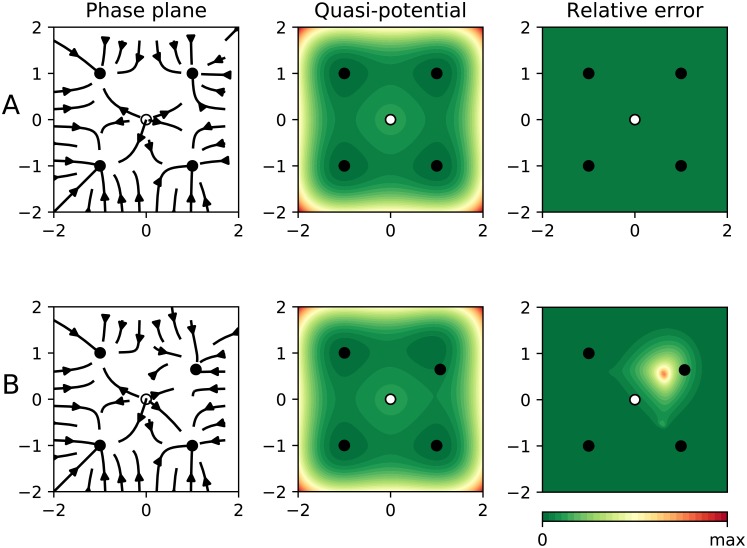
Results for two synthetic examples. In all panels the dots represent equilibrium points (black for stable, otherwise white). The left panel shows the phase plane containing the actual “deterministic skeleton” of the system. The central panel shows the quasi-potential. The right panel shows the estimated error. **Row A** shows the application to a gradient case (Eq (20) with interaction terms equal to zero). As expected, the error is zero everywhere. In **row B** our algorithm is applied to a non-gradient case (Eq (20), with non-zero interaction terms).

When we choose those non-gradient interactions to be zero, the system is purely gradient and corresponds with a four-well potential. Our algorithm rendered it successfully and with zero error (cf. Fig 4, row A). In order to test our algorithm, we introduced a non-gradient interaction of the form *p*_*x*_(*x*, *y*) = 0.3*y* ⋅ *m*(*x*, *y*) and *p*_*y*_(*x*, *y*) = −0.4*x* ⋅ *m*(*x*, *y*), with $m(x,y)=e^{(x-1{)}^{2}+(y-1{)}^{2}}$. *m*(*x*, *y*) serves as a masking function guaranteeing that our interaction term will be negligible everywhere but in the vicinity of (*x*, *y*) = (1, 1). After introducing this non-gradient interaction a four-well potential is still recognizable (cf. Fig 4, row B). As expected, the error was correctly captured to be zero everywhere but in the region around (*x*, *y*) = (1, 1). The error map warns us against trusting the quasi-potential in the upper right region, and guarantees that elsewhere it will work fine. Notice that, accordingly, the upper right stable equilibrium falls slightly outside its corresponding well. The rest of equilibria, to the contrary, fit correctly inside their corresponding wells.

#### A fully non-gradient system

**Fig 5:**
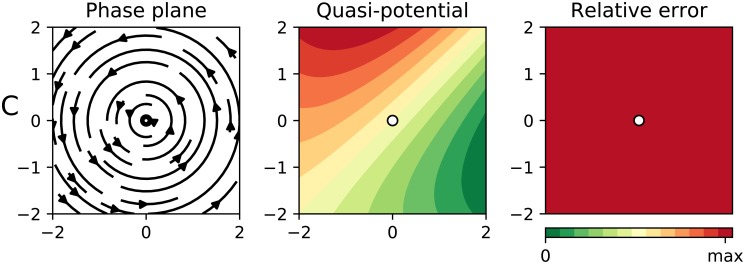
Results for a fully non-gradient system (Eq (21)). In all panels the dots represent center equilibrium points. The left panel shows the phase plane containing the actual “deterministic skeleton” of the system. The central panel shows the quasi-potential. The right panel shows the estimated error.

In order to stress our algorithm to the maximum, we tested it in a worst case scenario: a system with zero gradient part everywhere. Particularly, we fed it with Eq (21). All solutions (but the unstable equilibrium point at (0, 0)) are cyclic (cf. 5, left panel)). As we discussed in the introduction, calculating a potential for a system with cyclic trajectories is as impossible as Escher’s paintings (and for similar reasons). This fact is captured by our algorithm, that correctly predicts a relative error of 1 everywhere (see Fig 5, right panel). In this case, the quasi-potential (Fig 5, left panel) is not even locally useful.

$$\left\{ \begin{array}{c} \frac{dx}{dt}=-y\\ \frac{dy}{dt}=x \end{array}\right.$$ (21)

### Biological examples

#### A simple regulatory gene network

Waddington’s epigenetic landscapes [5, 22] are a particular application of stability landscapes to gene regulatory networks controlling cellular differentiation. When applied to this problem, stability/epigenetic landscapes serve as a visual metaphor for the branching pathways of cell fate determination.

A bistable network cell fate model can be described by a set of equations like (22). Such a system represents two genes (*x* and *y*) that inhibit each other. This circuit works as a toggle switch with two stable steady states, one with dominant *x*, the other with dominant *y* (see [23]).

$$\left\{ \begin{array}{c} \frac{dx}{dt}=b_{x}-r_{x}x+\frac{a_{x}}{k_{x}+y^{n}}\\ \frac{dy}{dt}=b_{y}-r_{y}y+\frac{a_{y}}{k_{y}+x^{n}} \end{array}\right.$$ (22)

**Fig 6:**
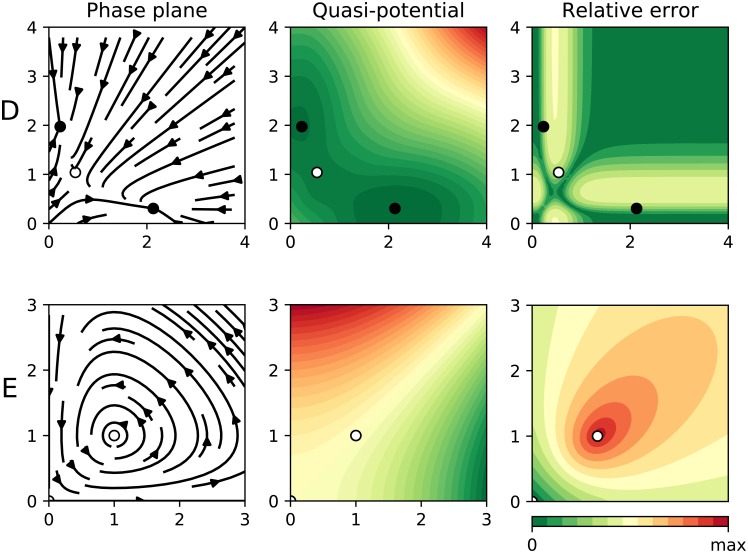
Results for two biological systems. In all panels the dots represent equilibrium points (black for stable, otherwise white). The left panel shows the phase plane containing the actual “deterministic skeleton” of the system. The central panel shows the quasi-potential. The right panel shows the estimated error. In **row D** we applied our algorithm to the simple gene regulatory network described in Eq (22). In **row E** we apply our algorithm to a Lotka-Volterra system (Eq (23), with *a* = *b* = *c* = *d* = 1).

Our parameter choice (*a*_*x*_ = 0.4802, *a*_*y*_ = 0.109375, *b*_*x*_ = 0.2, *b*_*y*_ = 0.3, *k*_*x*_ = 0.2401, *k*_*y*_ = 0.0625, *r*_*x*_ = *r*_*y*_ = 1 and, *n* = 4) corresponds with equations 6 and 7 of [23], where we modified two parameters (*By* = 0.3, *foldXY* = 1.75, in their notation) in order to induce an asymmetry in the the dynamics. Although this system is non-gradient, our algorithm correctly yields a pseudopotential with two wells (see Fig 6, row D). Observe that the bottom of those potentials corresponds with a stable equilibrium. The relative error, despite being distinct from zero in some regions, is not very high. This means that our quasi-potential is a reasonable approximation of the underlying dynamics. Indeed, the equilibria correspond to the wells (stable) and the peak (unstable).

#### Predator prey dynamics

The Lotka-Volterra model (Eq (23)) is a classical predator-prey model [24]. In this model *x* and *y* represent, respectively, the prey and predator biomasses.

$$\left\{ \begin{array}{c} \frac{dx}{dt}=ax-bxy\\ \frac{dy}{dt}=cxy-dy \end{array}\right.$$ (23)

This model is known to have cyclic dynamics. As we discussed in our analogy with Escher’s paintings, we cannot compute stability landscapes in the regions of the phase plane where the dynamics are cyclic. When we apply our method to a system like Eq (23), the error map correctly captures the fact that our estimated potential is not trustworthy (see Fig 6, row E).

#### Selkov model for glycolysis

The Selkov model for glycolysis reads like Eq (24), where *x* and *y* represent the concentrations of two chemicals.

$$\left\{ \begin{array}{cc} \frac{dx}{dt} & =-x+ay+x^{2}y\\ \frac{dy}{dt} & =b-ay-x^{2}y \end{array}\right.$$ (24)

**Fig 7:**
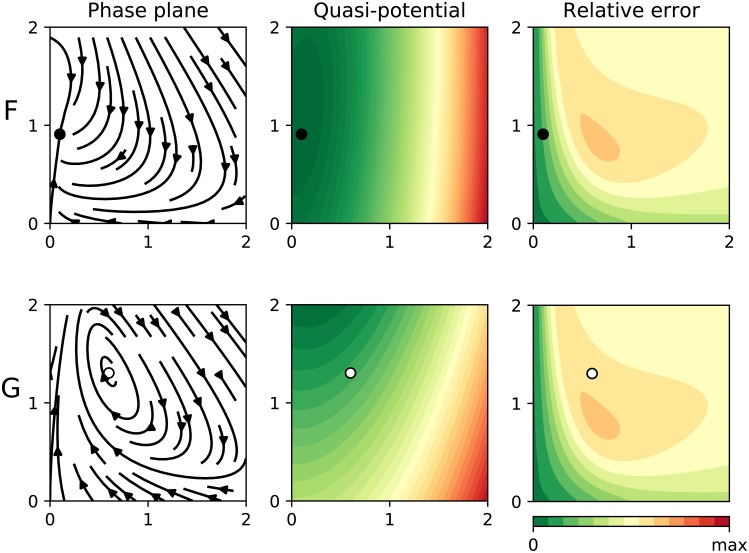
Results for two parameter settings of the Selkov model (Eq (24)). In all panels the dots represent equilibrium points (black for stable, otherwise white). The left panel shows the phase plane containing the actual “deterministic skeleton” of the system. The central panel shows the quasi-potential. The right panel shows the estimated error. In **row F** we applied our algorithm to a Selkov system with *b* = 0.1, so the solutions reach a stable point. In **row G** we set *b* = 0.6, so the system has a limit cycle.

The Selkov model is a classical example of a dynamical equation motivated by a biochemical problem that can develop a limit cycle under certain circumstances. Particularly, if we fix *a* = 0.1, such a system is known to have a limit cycle for *b* ∈ [0.42, 0.79] (Fig 7, row G), and otherwise it reaches an equilibrium solution (Fig 7, row F). This system is particularly interesting from the pedagogical point of view because the Jacobian doesn’t depend on *b*, so the error map remains the same.

In the configuration showed in row F, we see that the trajectories corresponding to low concentrations of any of both chemicals (i.e.: *x* ≪ 1 or *y* ≪ 1) remain in the “safe zone” according to the error map. Our estimated potential can be used in that region and, accordingly, the equilibrium point lies in the bottom of a potential well. On the other hand, in the configuration showed in row G, even those trajectories that start inside the “safe zone” are forced to “explore” the “unsafe” area outside it. Such a configuration leads to a limit cycle and thus, unsurprisingly, doesn’t admit a potential representation.

## Discussion

The use of stability landscapes as a helping tool to understand one-dimensional dynamical systems achieved great success, especially in interdisciplinary research communities. A generalization of the idea of scalar potential to two-dimensional systems seemed to be a logical next step. Unfortunately, as we have seen, there are reasons that make two (and higher) dimensional systems fundamentally different from the one-dimensional case. The generalization, straightforward as it may look, is actually impossible for most dynamical equations. A good example of this impossibility is any system with cyclic dynamics, whose scalar potential should be as impossible as the Penrose stairs in Escher’s paintings. As a consequence, any attempt of computing stability landscapes for non-gradient systems should, necessarily, drop some of the desirable properties of classical scalar potentials.

For instance, the method proposed by Bhattacharya [23] smartly tries to avoid the fundamental problem of path dependence of line integrals by integrating along trajectories, removing thus the freedom of path choice. A problem of this approach is that there is generally no continuity along the borders of the basins of attraction in the resulting quasi-potential, and that the results depend on the choice of the initial point. Our approach is, in some sense, the opposite. We embrace the fact that some systems do not have a reliable scalar potential landscape in some (or even all) regions of the phase space, and we show this fundamental limitation explicitly via the error map.

Methods based on probabilistic considerations such as occupancy probabilities at the steady-state distribution [16, 25] or transition rates between states [12, 14, 15, 17], provide a continuous landscape where deep areas correspond with states with a high probability of occurrence. When applied to a limit cycle, they give rise to “Mexican hat” shaped surfaces [16]. However, these surfaces cannot be interpreted straightforwardly as classical potentials where the state just “slides” to the bottom of the wells. That is, the “rolling-marble” analogy has to be used very carefully. Two transparent examples appear in fig. 3A in [16] or fig. 2B in [25]. While the slope correctly captures the attracting property towards the limit cycle, once inside of it the trajectories eventually go uphill. Additionally, these methods require familiarity with advanced mathematical concepts such as stochastic and partial differential equations, both of them more complex and computationally expensive than our approach.

We share the perception with other authors [4] that the concept of potential is often misunderstood in research communities with a limited mathematical background. The algorithm we introduce here is an attempt to preserve as much as possible from the classical potential theory while addressing explicitly its limitations and keeping the mathematical complexity as low as possible. A more detailed summary about what our algorithm provides is:

- Integrity. At each step the strength of the non-gradient term is calculated. If this term is high, it is fundamentally impossible to calculate a scalar potential with any method. If this term is zero, our solution converges to the classical potential.
- Safety. The relative size of the non-gradient term can be interpreted as an error term, mapping which regions of our stability landscape are dangerous to visit.
- Speed. The rendering of a printing quality surface can be performed in no more than a few seconds in a personal laptop.
- Simplicity. The required mathematical background is covered by any introductory course in linear algebra and vector calculus.
- Generality. The core of the algorithm is the skew-symmetric decomposition of the Jacobian. This operation can be easily applied to square matrices of any size, generalizing our algorithm for working in 3 or more dimensions.
- Usability. We provide the algorithm in the form of a ready to use, fully documented and tested *R* package [20].

It is important to notice that, although our algorithm provides us with a way of knowing which regions of the phase plane can be “safely visited”, we cannot navigate the phase plane freely but only along trajectories. This interplay between regions and trajectories limits the practical applicability of our algorithm to those trajectories that never enter regions with high error (good examples of this can be found in Fig 4 row B and Fig 7 row F). If our algorithm works in the region of interest, there is no need to use more advanced (and thus, more difficult to implement and to interpret) methods, as we expect them to converge to the same solution. As a rule of thumb, we found that any relative error below 0.2 can be considered small for visualization purposes, but this is a subjective criterion. For higher errors, or in case of doubt, we suggest to visually compare the calculated quasi-potential with the phase plane (as we did in all the figures in the Results section). If required, a more detailed assessment of the absolute error can be performed by calculating the difference between the flow containing the original dynamical equations and the flow corresponding to the quasi-potential (i.e., by direct evaluation of $\vec{f}(\vec{x})-{\vec{f}}_{g}(\vec{x})$, see Methods section). Note that the absolute error should not be calculated using the quasi-potential itself, but the flows derived from it.

If we conclude that the system doesn’t admit our quasi-potential, then the most reasonable alternative is to use a Freidlin-Wentzell potential, but keeping in mind that its interpretation is not so straightforward as that of a scalar potential.

The concept of potential is paramount in physical sciences. The main reason for the ubiquity of potentials in physics is that several (idealized) physical systems are known to be governed only by gradient terms (e.g.: movement in friction-less systems, classical gravitatory fields, electrostatic fields, …). As physical potentials can be related with measurable concepts like energy, its use goes way further than visualization. From the depth and width of a potential we can learn about transition rates and resilience to pulse perturbations. The height and shape of the lowest barrier determines the minimum energy to transition to an alternative stable state, which relates to the probability of a noise-driven jump in a stochastic environment [12, 18, 26]. All these results remain true for non-physical problems that happen to be governed exclusively by gradient dynamics, and, we claim, should remain approximately true for problems governed by weakly non-gradient dynamics. This is the situation our algorithm has been designed to deal with.

Regarding visualization alone, it may be worth reconsidering why we prefer the idea of stability landscape over a traditional phase plane figure, especially after pointing out all the difficulties of calculating stability landscapes for higher-dimensional systems. It is true that the phase plane is slightly less intuitive than the stability landscape, but it has a very desirable property: it doesn’t require the imagination of a surrealist artist to exist.

## Supporting information

S1 Text Appendix contaning some mathematical details. (PDF)
