# Supplementary material for: Climbing Escher’s stairs: A way to approximate stability landscapes in multidimensional systems
Source: PLoS Comput Biol. 2020 Apr 10;16(4):e1007788. doi: 10.1371/journal.pcbi.1007788 (PMC7176285; doi:10.1371/journal.pcbi.1007788)
Supplement: S1 Text — (PDF) [file pcbi.1007788.s001.pdf]

Additional information

Here we present some additional information for those readers interested in the mathematical details of the "Climbing Escher's stairs: a way to approximate stability landscapes in multidimensional systems".

Gradient conditions for a system with an arbitrary number of dimensions

Dynamics in equation (2) and the condition for the crossed derivatives (3) can be straightforwardly generalized (see equations (A.2) and (A.1)) to systems with an arbitrary number of state variables $\vec{x} = (x_1, ..., x_n)$. Particularly, if and only if our system of equations $\frac{dx_i}{dt} = f_i(\vec{x})$ satisfies the condition for all i:

$$\frac{\partial f_i}{\partial x_j} = \frac{\partial f_j}{\partial x_i} : i \neq j \tag{A.1}$$

then a potential $V(\vec{x})$ exists related to the original vector field:

$$\frac{dx_i}{dt} = f_i(\vec{x}) = -\frac{\partial V}{\partial x_i} : i = 1..n \tag{A.2}$$

and such a potential can be computed using a line integral:

$$V(\vec{x}) = V(\vec{x}_0) - \int_\Gamma \sum_{i=1}^{n} f_i(\vec{x}) dx_i \tag{A.3}$$

where the line integral in (A.3) is computed along any curve Γ joining the points $\vec{x}_0$ and $\vec{x}$.

It is important to note that the number of equations (N) described in condition (A.1) grows rapidly with the dimensionality of the system (D), following the series of triangular numbers $N = \frac{1}{2}(D-1)D$. Thus, the higher the dimensionality, the harder it may get to fulfill condition (A.1). As a side effect, we see that one-dimensional systems have zero conditions and their stability landscape is thus always well-defined.

Correspondence with the Helmholtz decomposition

Our decomposition (15) is an approximation of the Helmholtz decomposition. The Helmholtz decomposition is defined as the decomposition of the field in a gradient term and a curl, or divergence-free term. This decomposition is known to be unique.

The gradient nature of $\vec{f}_g(\vec{x})$ has already been established in the Methods section. Thus, in order to prove the correspondence, we only need to show that $\vec{f}_{ng}(\vec{x})$ is a divergence-free field, that is, $\vec{\nabla} \cdot \vec{f}_{ng} = 0$. The divergence represents one of the many generalizations of the concept of derivative in systems with 2 and more dimensions, and it is a central concept from vector calculus [21]. The divergence operator $\vec{\nabla}\cdot$ of a field in cartesian coordinates is defined as the sum of the derivative of each element respective the corresponding coordinate (see equation (A.4) for an example using the two-dimensional field $\vec{F}(x,y) = (F_x(x,y), F_y(x,y))$).

$$\vec{\nabla} \cdot \vec{F} = \frac{\partial F_x}{\partial x} + \frac{\partial F_y}{\partial y} \tag{A.4}$$

When applied to $\vec{f}_{ng}$, defined as in equation (15), the divergence equals the sum of the diagonal elements of J_{skew}. The diagonal elements of any skew matrix are all zero (see (8)), and thus, the divergence of $\vec{f}_{ng}$ is zero too.

Detailed example of application

To calculate the value of V at, for instance, the point (x_3, y_2) of a grid, we should begin by assigning 0 to the potential at our arbitrary starting point (i.e.: $V(x_0, y_0) = 0$ by definition). Then, we need a trajectory that goes from (x_0, y_0) to (x_3, y_2), iterating over the intermediate grid points (see figure A.1).

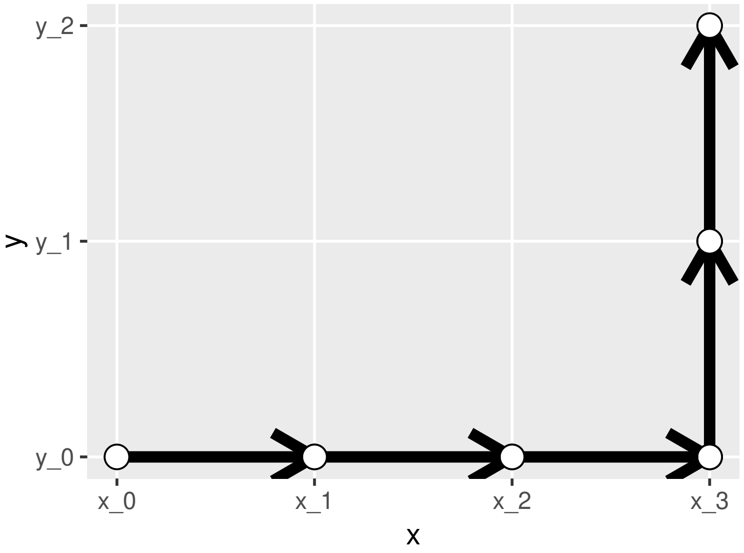

Figure A.1: Path used to go from point (x_0, y_0) to (x_3, y_2). Note that this is not the only possible path. Our algorithm converges to the same potential regardless of the path chosen thanks to neglecting the skew part of the Jacobian in our linearization process.

In the first step we go from (x_0, y_0) to (x_1, y_0). The new potential is thus (using (17)):

$$V(x_1, y_0) \approx V(x_0, y_0) + \Delta V(x_1, y_0; x_0, y_0)$$

The next two steps continue in the horizontal direction, all the way to (x_3, y_0). The value of the potential there is:

$$V(x_3, y_0) \approx V(x_0, y_0) + \Delta V(x_1, y_0; x_0, y_0) + \Delta V(x_2, y_0; x_1, y_0) + \Delta V(x_3, y_0; x_2, y_0)$$

Now, to reach our destination (x_3, y_2) we have to move two steps in the vertical direction:

$$V(x_3, y_2) \approx V(x_0, y_0) + \Delta V(x_1, y_0; x_0, y_0) + \Delta V(x_2, y_0; x_1, y_0) + \Delta V(x_3, y_0; x_2, y_0) + \\ + \Delta V(x_3, y_1; x_3, y_0) + \Delta V(x_3, y_2; x_3, y_1)$$

Generalizing the previous example we see that we can compute the approximate potential at a generic point (x_i, y_j) using the closed formula (18). Both our example and formula (18) have been derived sweeping first in the horizontal direction and next in the vertical one. Of course, we can choose different paths of summation. Nevertheless, because we are building our potential neglecting the non-gradient part of our vector field, we know that our results will converge to the same solution regardless of the chosen path.
